# Incompatible amount of 3-D and 2-D periodontal attachments on micro-CT scanned premolars

**DOI:** 10.1371/journal.pone.0193894

**Published:** 2018-03-08

**Authors:** Hsiang-Hsi Hong, Adrienne Hong, Yi-Fang Huang, Heng-Liang Liu

**Affiliations:** 1 Department of Periodontics, Chang Gung Memorial Hospital and Chang Gung University, Linkou, Taiwan; 2 School of Dental Technology, College of Oral Medicine, Taipei Medical University, Taipei, Taiwan; 3 California North State University, College of Medicine, Elk Grove, California, United States of America; 4 Department of General Dentistry, Chang Gung Memorial Hospital, Linkou, Taiwan; 5 Instrument Department, Chang Gung Memorial Hospital, Linkou, Taiwan; University of Tennessee Health Science Center, UNITED STATES

## Abstract

Micro-computed tomography (micro-CT) was employed to relate the root surface area (RSA) to the periodontal attachment levels (PALs) of extracted premolars to diagnose periodontitis. Single-rooted human maxillary and mandibular premolars 31 and 36, respectively, were surveyed by micro-CT and its associated software. RSA levels from the 1^st^ to 10^th^ mm, corono-apically, were analyzed using statistical *t* tests. The average root length (RL) and RSA of the maxillary and mandibular premolars were significantly different (*p* < 0.05). Both premolars demonstrated a non-significant RSA percentage comparison at the evaluated PALs. For the 30% coronal 2-D radiographic RL, the 3-D RSAs 3.77 mm and 3.99 mm apical to the cementoenamel junction (CEJ) were 39.48% and 40.65% for maxillary and mandibular premolars, respectively. At the 15% coronal 2-D RL, the 3-D RSA 2 mm apical to the CEJ of the premolars was approximately 21%. At the 50% coronal 2-D RL level, approximately 62% coronal 3-D RSA and 6.5 mm RL decreased. The amount of decrease of the RSA attachment is significant in every 2-mm measurement for both premolars. Sampling periodontal microbial pathogens based on the condition of 2-D radiographic bone and clinical attachment losses without considering 3-D RSA is potentially inadequate and may underestimate the severity of the periodontitis.

## Introduction

Periodontists rely greatly on the quantity and quality of the remaining periodontal attachment for diagnosis, classification, treatment plans and prognosis of periodontitis. Clinical attachment loss (CAL) and radiographic bone loss are important parameters that help indicate the severity of periodontitis and the results of the related periodontal treatment. According to the 2015 American Academy of Periodontology (AAP) guidelines, mild periodontitis is characterized by losing 1 to 2 mm CAL and 15% radiographic bone; moderate periodontitis involves a loss of 3 to 4 mm CAL and 16% to 30% radiographic bone; and in severe periodontitis, there is a loss of ≥5 mm CAL and >30% radiographic bone [1]. However, the probing attachment level offers the reference of CAL in a one-dimensional measurement without taking the root shape and root length into account, which underestimates the amount of true periodontal attachment loss [2]. The limitations of measuring probing pocket depth in evaluating the clinical attachment level are also well-recognized [3]. Exploration of the association between the root surface area and periodontal attachment has been undertaken using various tooth types [4–7] and study methods [8,9].

Human premolars display a taper and a complicated root anatomy (i.e., wider faciolingual distribution than mesiodistal distribution at different cross-sectional levels from the cementoenamel junction (CEJ) to the apex), and the differentiation in the percentage of maximal periodontal attachment evaluated using a 2-D standpoint from a 3-D standpoint is still limited [10].

Approximately 300 bacterial species have been identified and found to contribute to periodontal pocket dental biofilm. Although only a small number of bacterial species are related to severe periodontitis, these periodontal pathogens have been discussed based on the 2-D perspective approach [1,11].

Currently, three-dimensional techniques are extensively applied in dental practice. A new generation of micro-computed tomography (micro-CT) scan uses micro focal spot X-ray sources and high-resolution detectors, allowing for projections that can be rotated through multiple viewing directions to produce three-dimensional reconstructed images of samples, and can reach a spatial resolution of 15–18 μm, which corresponds to nearly 3x10^-6^ cubic mm in voxel size. The images represent spatial distribution maps of linear attenuation coefficients that are determined by the energy of the X-ray source and the atomic composition of the material sample. Since the imaging process is nondestructive, the internal features of the same sample may be examined many times, and samples remain available after scanning for additional biological and mechanical testing. Micro-CT systems are now available in dental academic fields; several studies have provided reviews and analysis of micro-CT imaging [10–14]. Through software programs, the scanned 3-D files can be translated into 2-D data for the root surface area (RSA) analysis. The relationship between theroot surface area (RSA) and CAL can then be established. However, studies that use micro-CT to inspect CAL are still limited. It was hypothesized that the decreasing amounts and ratios of periodontal attachment at levels examined from the CEJ to the apex that were calculated via 3-D RSA are different from those that were evaluated by 2-D radiographic evaluations. The aims of this study were to use micro-CT to measure the RSA amount and the ratio of periodontal attachment from the levels of the CEJ to the apexin1 to 10 millimeters and to evaluate the various RSA amounts and ratios between two subsequent levels of extracted premolars. The RSAs corresponding to periodontal attachment levels in mild–severe periodontitis were also explored.

## Materials and methods

Thirty-one maxillary and 36 mandibular intact human premolars were extracted and collected from patients who underwent orthodontic or periodontal treatment at the Dental Department of the Chang Gung Memorial Hospital. All of the patients were aged 18 to 65 and provided written informed consent. The extracted teeth were stored in 10% formalin and were subsequently washed under tap water. Soft tissues, calculus and stains were removed carefully using an ultrasonic scaler and a curette. Teeth were autoclaved before being scanned. The study protocol was approved by an Institutional Review Board for Clinical Research in CGMH.

**Fig 1:**
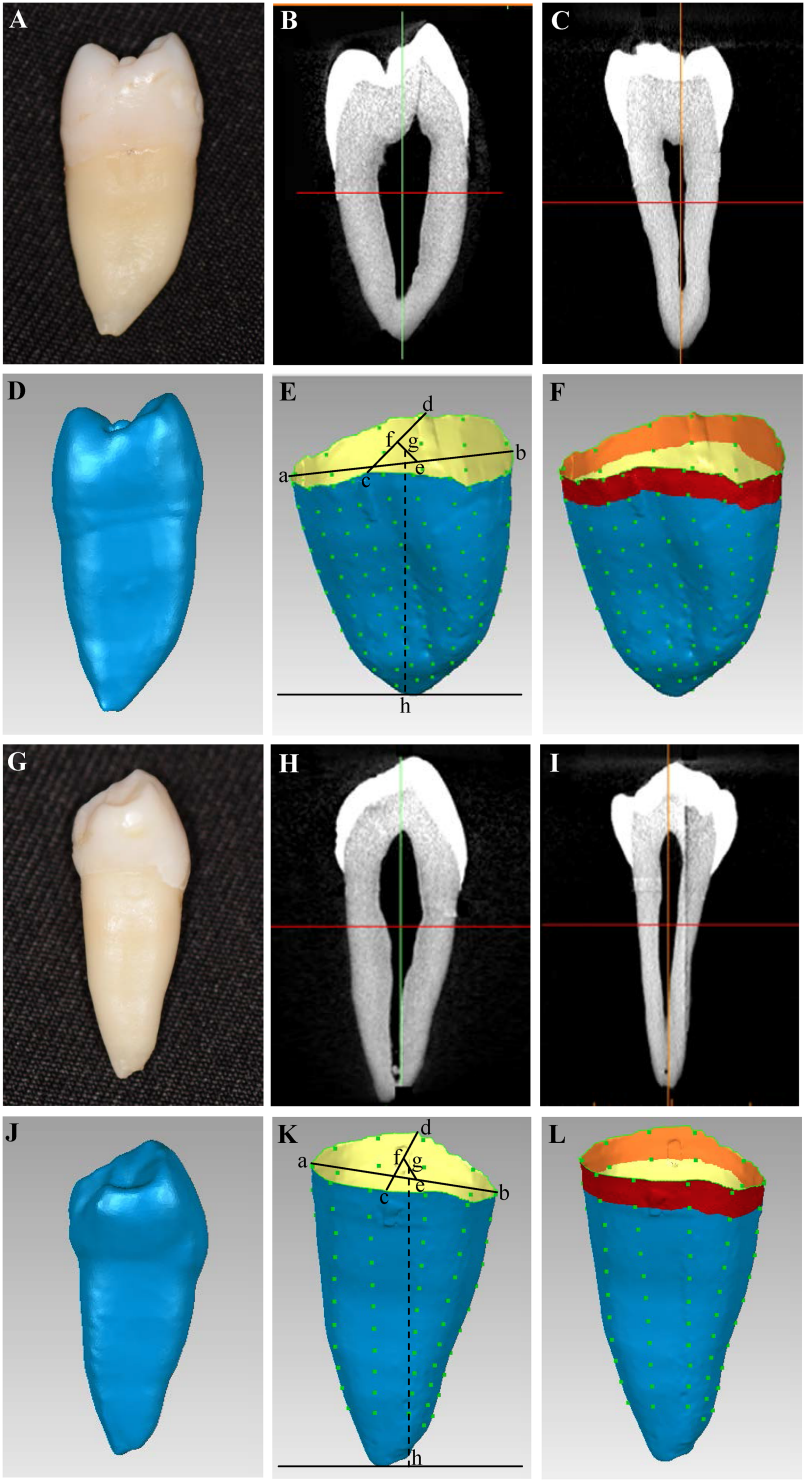
Photograph, micro-CT and STL images of scanned maxillary and mandibular premolars. (A–F): The views and estimated levels of a micro-CT scanned maxillary premolar. (A): maxillary premolar; (B): a sagittal view; (C): a frontal view; (D): a STL format model was developed.; (E): a–b: line connecting the buccal and lingual CEJ, c–d: line connecting the mesial and distal CEJ, e: midpoint of a–b, f: midpoint of c–d, g: midpoint of e–f and represented the CEJ from 2–D viewpoint, and g–h: represented the 2–D root length; (F): The root surface areas were calculated from 1^st^ mm to 10^th^ mm. (G–L): The views and evaluated levels of a micro-CT scanned mandibular premolar. (G): mandibular premolar; (H): a sagittal view; (I): a frontal view; (J): a STL format model was developed; (K): a–b: line connecting the buccal and lingual CEJ, c–d: line connecting the mesial and distal CEJ, e: midpoint of a–b, f: midpoint of c–d, g: midpoint of e–f and represented the CEJ from 2–D viewpoint, and g–h: represented the 2–D root length; (L): The root surface areas were calculated from 1^st^ mm to 10^th^ mm corono-apically.

Information on the external and internal structure of the specimens were obtained using micro-computed tomography (micro-CT; SkyScan 1076, Bruker, Kontich, Belgium) by rotating the premolars along the tooth axis and scanning the teeth from the incisal edge to the root apex. The micro-CT was set to the following conditions: pixel matrix, 2,000×2,000; tube voltage, 100 kV; tube current, 100 μA; slice thickness, 18 μm. The apex of each premolar was fixed vertically on a fixture parallel to the long axis of the tooth root. The scanning time of each tooth was approximately 10 min. Data were exported from the micro-CT machine in DICOM (Digital Imaging and Communications in Medicine) and TIF (Tagged Image File) data format. DataViewer and CTVox software (Bruker, Kontich, Belgium) were used to examine the structure of the sample. Two-dimensional images were used to generate three-dimensional reconstructions with the CTAn software (Bruker, Kontich, Belgium). Three-dimensional geometry files were reconstructed for each mask and saved as stereolithography (STL) files. An STL format model was developed with approximately 1,500,000–2,000,000 fine triangle surfaces for describing three-dimensional premolars (Fig 1D and 1J). The premolar root surface areas were calculated and analyzed as the sum of specific fine triangle areas using the Pro/ENGINEER software (PTC, Needham, MA, USA). The 2-D root length was decided by connecting the root apex and the midpoint (2-D CEJ) between interproximal and buccolingual cross lines, which were used as the landmarks to define the root length for a tooth (Fig 1E and 1K). The 3-D CEJ was measured by connecting the marked CEJs at 15 to 20 sagittal, horizontal and frontal sections (Fig 1B, 1C, 1F, 1H, 1I and 1L). The RSA amount and percentage at 1^st^mm, 2^nd^mm, 3^rd^mm, 4^th^mm, 5^th^mm, 6^th^mm, 7^th^mm, 8^th^mm, 9^th^ mm, and 10^th^ mm periodontal attachment levels (PAL) from the CEJ to the apex of the maxillary and mandibular premolars were calculated and analyzed (Fig 1F and 1L)(S1 Table). We also assessed the associated RSA amount and percentage with the coronal 15%, 30% and 50% radiographic bone loss (RBL) at the 2-D radiographic X-ray viewpoint.

### Statistical analysis

Both maxillary and mandibular premolars were statistically analyzed after determining the ratio of the RSA at planned levels to the total RSA for every individual tooth from the 1^st^ to 10^th^ mm first before intra- or inter-groups analyses to avoid tooth size and root morphology bias. Significant differences between maxillary and mandibular samples, as well as the significance between the tested intra-group levels, were investigated using repeated-measures analysis of variance (ANOVA), paired *t* test, independent *t* test and one-sample *t* test.

A repeated-measures ANOVA was used to assess whether the relative RSA amount and percentages at the evaluated PALs fell within the 95% confidences intervals (CI, *p*< 0.05).

Paired *t* tests were employed to explore the significance of the RSA amount and percentages between two subsequent surveyed levels from the CEJ to the 10^th^ mm corono-apically of the RSA (*p*< 0.05).

Independent *t* tests were utilized to study the RSA variation at corresponding levels of maxillary and mandibular premolars (*p*< 0.05).

A one-sample *t* test was applied to compare the RSA differences between 3-D and 2-D perspectives at 15%, 30% and 50% RBLs (*p*< 0.01).

The null hypothesis of this test (H0) was that the RSA variation between 3-D and 2-D viewpoints at 15%, 30% and 50% RBLs were ≤ 2% (|mean -15% (or 30%, or 50%)|≤ 2%).

The alternate hypothesis of this test (H1) was that the RSA variation between 3-D and 2-D viewpoints at 15%, 30% and 50% RBLs were >2% (|mean—15% (or 30%, or 50%)| >2%).

## Results

The images of tooth cross-sections were combined and converted to a 3-D structure (Fig 1). The evolved 2-D RSAs from 3-D root scans were measured and analyzed.

The results revealed that the average root lengths/RSA of the maxillary and mandibular premolars were 12.63 ± 0.19 mm (range from 10.7 mm to 14.4 mm)/226.16 ± 5.99 mm^2^ (range from 170.2 mm^2^ to 299.5 mm^2^) and 13.3 ± 0.26 mm (range from 10.3 mm to 17.0 mm)/202.41± 4.25 mm^2^ (range from 161.2 mm^2^ to 272.0 mm^2^), respectively. Both the RSA and the root length of the premolars showed a wide distribution range; in addition, maxillary and mandibular premolars revealed a significant variation in RSA and root length comparisons (*p* < 0.05). However, repeated-measures analysis of variance (ANOVA) revealed that all of the decreases in RSA percentages apical to the CEJ at surveyed PALs fell into the 95% CI for both premolars. In addition, maxillary and mandibular premolars demonstrated non-significant differences in the decrease in RSA percentages at the evaluated PALs in millimeters corono-apically (Table 1).

**Table 1:** RSA amount and percentages at the evaluated PALs corono-apically.

	Maxillary premolars (n = 31)		Mandibular premolars (n = 36)		Maxilla vs. Mandible
**RSA at various PALs**	Average ± SE	95% CI	Average ± SE	95% CI	*P* < 0.05
RSA at 100% PAL	226.16 ± 5.99 mm^2^		202.41 ± 4.25 mm^2^		*P* = 0.002**
Root length	12.63 ± 0.19 mm		13.3 ± 0.26 mm		*P* = 0.045*
Lost RSA mm^2^ & % at **1**^**st**^**mm** PAL	26.01 ± 0.49 mm^2^		23.45 ± 0.37 mm^2^		
	11.62 ± 0.22%	11.19~12.05	11.7 ± 0.22%	11.26~12.14	0.798
Lost RSA mm^2^ & % at **2**^**nd**^**mm** PAL	49.45 ± 0.96 mm^2^		43.74 ± 0.55 mm^2^		
	22.10 ± 0.43%	21.26~22.94	21.83 ± 0.37%	21.10~22.56	0.643
Lost RSA mm^2^ & % at **3**^**rd**^**mm** PAL	73.74 ± 1.32 mm^2^		63.00 ± 1.57 mm^2^		
	32.96 ± 0.60%	31.79~34.12	31.48 ± 0.46%	30.57~32.39	0.052
Lost RSA mm^2^ & % at **4**^**th**^**mm** PAL	95.67 ± 1.61 mm^2^		82.05 ± 0.98 mm^2^		
	42.76 ± 0.76%	41.27~44.26	40.90 ± 059%	39.73~42.06	0.055
Lost RSA mm^2^ & % at **5**^**th**^**mm** PAL	116.39 ± 1.95 mm^2^		100.26 ± 1.51 mm^2^		
	52.00 ± 0.86%	50.31~53.68	49.88 ± 0.66%	48.58~51.17	0.052
Lost RSA mm^2^ & % at **6**^**th**^**mm** PAL	136.29 ± 2.26 mm^2^		117.00 ± 1.68 mm^2^		
	60.86 ± 0.93%	59.04~62.68	58.22 ± 0.78%	56.70~59.75	0.032
Lost RSA mm^2^ & % at **7**^**th**^**mm** PAL	154.31 ± 3.02 mm^2^		133.59 ± 1.93 mm^2^		
	68.76 ± 1.02%	66.77~70.76	66.46 ± 0.88%	64.68~68.26	0.091
Lost RSA mm^2^ & % at **8**^**th**^**mm** PAL	171.96 ± 3.37 mm^2^		149.29 ± 2.31 mm^2^		
	76.59 ± 1.05%	74.53~78.66	74.23 ± 0.96%	72.35~76.12	0.103
Lost RSA mm^2^ & % at **9**^**th**^**mm** PAL	188.53 ± 3.86 mm^2^		163.62 ± 2.70 mm^2^		
	83.90 ± 1.07%	81.80~85.99	81.27 ± 0.95%	79.42~83.13	0.070
Lost RSA mm^2^ & % at **10**^**th**^**mm** PAL	201.16 ± 4.28 mm^2^		177.25 ± 3.38 mm^2^		
	89.42 ± 0.96%	87.54~91.30	87.90 ± 0.94%	86.05~89.75	0.265

Repeated measures analysis of variance (RMAV) revealed if the lost amount and percentages of RSA at surveyed PALs are in 95% confidence intervals (CI).

Independent *t* test for maxilla vs. mandible:

*: *p*< 0.05,

**: *p*< 0.01

PAL: Periodontal attachment level measured from CEJ to root apex

RSA: Root surface area with periodontal attachment determined by a 3-D image.

Corono-apically, maxillary and mandibular premolar roots retained 11.6%and 11.7% RSA at the 1^st^ mm PAL, 10.48% and 10.13% RSA at the 2^nd^ mm PAL, 10.86% and 9.65% RSA at the 3^rd^ mm PAL, 9.81% and 9.41% RSA at the 4^th^ mm PAL, 9.23% and 8.98% RSA at the 5^th^ mm PAL, 8.86% and 8.34% RSA at the 6^th^ mm PAL, 7.91% and 8.24% RSA at the 7^th^ mm PAL, 7.83% and 7.77% RSA at the 8^th^ mm PAL, 7.30% and 7.04% RSA at the 9^th^ mm PAL and 5.52% and 6.63% RSA at the 10^th^ mm PAL apical to CEJ, respectively. Mostly, maxillary and mandibular premolars demonstrated insignificant differences in RSA percentages/mm at corresponding levels apical to CEJ (*p*> 0.05, Table 2).

**Table 2:** Amount and percentages of RSAs at each PALs corono-apically.

RSA at various PALs	Maxillary premolars (n = 31)	Mandibular premolars (n = 36)	Maxilla vs. Mandible
	Average ± SE	Average ± SE	P< 0.05 (%)
**RSA mm**^**2**^ **& % from** 0 to 1^st^ **mm PAL**	26.01 ±0.49 mm^2^	23.45 ±0.37 mm^2^	
	11.62 ± 0.22%	11.70 ± 0.22%	0.798
**RSA mm**^**2**^ **& % from** 1^st^ to 2^nd^ **mm PAL**	23.44 ± 0.57mm^2^	20.30 ± 0.26 mm^2^	
	10.48 ± 0.25%	10.13 ± 0.18%	0.254
**RSA mm**^**2**^ **& % from** 2^nd^ to 3^rd^**mm PAL**	24.29 ± 0.65 mm^2^	19.26 ± 1.39 mm^2^	
	10.86 ± 0.30%	9.65 ± 0.24%	0.003*
RSA mm^2^ & % from 3^rd^ to 4^th^ **mm PAL**	21.92 ± 0.47 mm^2^	19.05 ± 1.29 mm^2^	
	9.81 ± 0.23%	9.41 ± 0.18%	0.184
**RSA mm**^**2**^ **& % from** 4^th^ to 5^th^ **mm PAL**	20.73 ± 0.73 mm^2^	18.20 ± 0.64 mm^2^	
	9.23 ± 0.29%	8.98 ± 0.20%	0.461
**RSA mm**^**2**^ **& % from** 5^th^ to 6^th^**mm PAL**	19.90 ± 0.62 mm^2^	16.75 ± 0.41 mm^2^	
	8.86 ± 0.24%	8.34 ± 0.20%	0.100
**RSA mm**^**2**^ **& % from** 6^th^ to 7^th^ **mm PAL**	18.02 ± 1.27 mm^2^	16.58 ± 0.41 mm^2^	
	7.91 ± 0.57%	8.24 ± 0.18%	0.549
**RSA mm**^**2**^ **& % from** 7^th^ to 8^t**h**^ **mm PAL**	17.65 ± 0.72 mm^2^	15.71 ± 0.42 mm^2^	
	7.83± 0.28%	7.77 ± 0.13%	0.838
**RSA mm**^**2**^ **& % from** 8^th^ to 9^th^ **mm PAL**	16.57 ± 0.77 mm^2^	14.32 ± 0.59 mm^2^	
	7.30 ± 0.27%	7.04 ± 0.21%	0.435
**RSA mm**^**2**^ **& % from** 9^th^ to 10^t**h**^ **mm PAL**	12.63 ± 1.60 mm^2^	13.63 ± 0.80 mm^2^	
	5.52 ± 0.67%	6.63 ± 0.24%	0.108

Independent *t* test for maxilla vs. mandible:

*: *p*<0.01

Generally, the estimated levels displayed insignificant differences with subsequent mm apically for both maxillary and mandibular premolars (*p*> 0.05), except for the RSA of coronal 1^st^ mm vs. 2^nd^ mm PAL and 3^rd^ mm vs. 4^th^ mm PAL for maxillary premolars and 1^st^ mm vs. 2^nd^ mm, 5^th^ mm vs. 6^th^ mm, 7^th^ mm vs. 8^th^ mm and 8^th^ mm vs. 9^th^ mm PAL for mandibular premolars (*p*< 0.05, Table 3). However, with evaluation for every 2 mm, significant differences were found in all surveyed levels for both maxillary and mandibular premolars (*p*< 0.05, Table 3).

**Table 3:** Varied amount and percentages of RSA between two subsequent 1 mm and 2 mm PALs corono-apically.

	Maxillary premolars (n = 31)		Mandibular premolars (n = 36)	
RSA at various PALs	Average ± SE	*p*< 0.05	Average ± SE	*p*< 0.05
**varied RSA mm**^**2**^ **& % b/w** 1^st^& 2^nd^ mm **PAL**	26.01±0.49 mm^2^ vs. 23.44±0.57mm^2^		23.45±0.37 mm^2^ vs. 20.30±0.26mm^2^	
	11.62±0.22% vs. 10.48±0.25%	<0.001***	11.70±0.22% vs. 10.13±0.18%	<0.001***
**varied RSA mm**^**2**^ **& % b/w** 2^nd^& 3^rd^ mm **PAL**	23.44±0.57mm^2^ vs. 24.29±0.65 mm^2^		20.30±0.26mm^2^ vs. 19.26±1.39 mm^2^	
	10.48±0.25% vs. 10.86±0.30%	0.258	10.13±0.18% vs. 9.65±0.24%	0.085
**varied RSA mm**^**2**^ **& % b/w** 3^rd^& 4^th^ mm **PAL**	24.29±0.65 mm^2^ vs. 21.92±0.47 mm^2^		19.26±1.39 mm^2^ vs. 19.05±1.29 mm^2^	
	10.86±0.30% vs. 9.81±0.23%	<0.001***	9.65±0.24% vs. 9.41±0.18%	0.392
**varied RSA mm**^**2**^ **& % b/w** 4^th^& 5^th^ mm **PAL**	21.92±0.47 mm^2^ vs. 20.73±0.73 mm^2^		19.05±1.29 mm^2^ vs. 18.20±0.64 mm^2^	
	9.81±0.23% vs. 9.23±0.29%	0.075	9.41±0.18% vs. 8.98±0.20%	0.115
**varied RSA mm**^**2**^ **& % b/w** 5^th^& 6^th^ mm **PAL**	20.73±0.73 mm^2^ vs. 19.90±0.62mm^2^		18.20±0.64 mm^2^ vs. 16.75±0.41mm^2^	
	9.23±0.29% vs. 8.86±0.24%	0.304	8.98±0.20% vs. 8.34±0.20%	0.020*
**varied RSA mm**^**2**^ **& % b/w** 6^th^& 7^th^ mm **PAL**	19.90±0.62 mm^2^ vs. 18.02±1.27mm^2^		16.75±0.41 mm^2^ vs. 16.58±0.41mm^2^	
	8.86±0.24% vs. 7.91±0.57%	0.097	8.34±0.20% vs. 8.24±0.18%	0.631
**varied RSA mm**^**2**^ **& % b/w** 7^th^& 8^th^ mm **PAL**	18.02±1.27 mm^2^ vs. 17.65±0.72mm^2^		16.58±0.41 mm^2^ vs. 15.71±0.42mm^2^	
	7.91±0.57% vs. 7.83±0.28%	0.909	8.24±0.18% vs. 7.77±0.13%	<0.001***
**varied RSA mm**^**2**^ **& % b/w** 8^th^& 9^th^ mm **PAL**	17.65±0.72 mm^2^ vs. 16.57±0.77mm^2^		15.71±0.42 mm^2^ vs. 14.32±0.59mm^2^	
	7.83±0.28% vs. 7.30±0.27%	0.126	7.77±0.13% vs. 7.04±0.21%	0.006**
**varied RSA mm**^**2**^ **& % b/w** 9^th^& 10^th^ mm **PAL**	16.57±0.77 mm^2^ vs. 12.63±1.60mm^2^		14.32±0.59 mm^2^ vs. 13.63±0.80mm^2^	
	7.30±0.27% vs. 10.48±0.25%	0.050	7.04±0.21% vs. 6.63±0.24%	0.054
**Varied data between two subsequent 2mm PALs**				
**varied RSA mm**^**2**^ **& % b/w** 1^st^& 2^nd^ 2 mm **PAL**	49.45±0.96 mm^2^ vs. 46.22±0.99 mm^2^		43.74±0.55 mm^2^ vs. 38.31±0.72 mm^2^	
	22.10±0.43% vs. 20.67±0.48%	0.008**	21.83±0.37% vs. 19.06±0.34%	<0.001***
**varied RSA mm**^**2**^ **& % b/w** 2^nd^& 3^rd^ 2 mm **PAL**	46.22±0.99 mm^2^ vs. 40.63±1.09 mm^2^		38.31±0.72 mm^2^ vs. 34.95±0.82 mm^2^	
	20.67±0.48% vs. 18.09±0.39%	<0.001***	19.06±0.34% vs. 17.33±0.30%	<0.001***
**varied RSA mm**^**2**^ **& % b/w** 3^rd^& 4t^h^ 2 mm **PAL**	40.63±1.09mm^2^ vs. 35.67±1.56 mm^2^		34.95±0.82 mm^2^ vs. 32.29±0.78 mm^2^	
	18.09±0.39% vs. 15.74±0.59%	<0.001***	17.33±0.30% vs. 16.01±0.29%	<0.001***
**varied RSA mm**^**2**^ **& % b/w** 4^th^& 5^th^ 2 mm **PAL**	35.67±1.56 mm^2^ vs. 29.20±1.59 mm^2^		32.29±0.78 mm^2^ vs. 27.95±1.32 mm^2^	
	15.74±0.59% vs. 12.82±0.55%	<0.001***	16.01±0.29% vs. 13.66±0.41%	<0.001***

Paired *t* test for the RSA (%) significance at various PAL levels:

*: *p* < 0.05,

**: *p*< 0.01,

***: *p*< 0.001

At the coronal 15% 2-D radiographic bone level, the crestal position moved 1.89 mm, 47.23 mm^2^ and 20.99% RSA apically from the CEJ for maxillary premolars and 2.00 mm, 43.92 mm^2^ and 21.76% RSA for mandibular premolars. In correlation with the coronal 30% 2-D radiographic bone level, the maxillary alveolar crest shifted 3.77 mm, 89.09 mm^2^ and 39.47% RSA apically to the CEJ, with values of 3.99 mm, 82.12 mm^2^ and 40.65% RSA for mandibular premolars. At the stage of 50% 2-D radiographic bone loss, the maxillary alveolar crest was positioned 6.28 mm, 139.65 mm^2^ and 61.77% RSA apically to the CEJ, with values of 6.65 mm, 127.91 mm^2^ and 63.28% RSA for mandibular premolars (H1 and *p*< 0.05, Table 4). Insignificant maxillary and mandibular RSA percentage differences were seen at 15%, 30% and 50% RBL (*p*> 0.05, Table 4).

**Table 4:** The RSA amount, RSA percentages and root length at the evaluated RBLs corono-apically.

	Maxillary premolars (n = 31)		Mandibular premolars (n = 36)		Maxilla vs. Mandible
RBL %	Average ± SE	(*p*< 0.01)	Average ± SE	(*p*< 0.01)	P< 0.05
RSA at 0% RBL	226.16 ± 5.99 mm^2^		202.41 ± 4.25 mm^2^		0.002*
RSA at 0% RL	12.63 ± 0.19 mm		13.30 ± 0.26 mm		0.045*
RSA amount, percentage & RL at **15% BL**	47.23 ± 1.17 mm^2^		43.92 ± 0.95 mm^2^		
	20.99 ± 0.44%	vs. 15% RSA<0.001 H1	21.76 ± 0.31%	vs. 15% RSA<0.001 H1	0.150
	1.89 ± 0.03 mm		2.00 ± 0.04 mm		
Lost RSA amount, percentage & RL at **30% BL**	89.09 ± 2.23 mm^2^		82.12 ± 1.73 mm^2^		
	39.48 ± 0.64%	vs. 30% RSA<0.001 H1	40.65 ± 0.48%	vs. 30% RSA<0.001 H1	0.142
	3.77 ± 0.06 mm		3.99 ± 0.08 mm		
Lost RSA amount, percentage % & RL at **50%** BL	139.65 ± 3.56 mm^2^		127.91 ± 2.65 mm^2^		
	61.77 ± 0.81%	vs. 50% RSA<0.001 H1	63.28 ± 0.59%	vs. 50% RSA<0.001 H1	0.128
	6.28 ± 0.09 mm		6.65 ± 0.13 mm		

Independent *t* test for maxilla vs. mandible:

*: *p*< 0.05

RBL: Radiographic bone level evaluated from CEJ to apex at 2D standpoint

RL: Radiographic root length measured from CEJ to apex at 2D viewpoint

H1: One sample *t* test

## Discussion

The concept of clinical attachment loss (CAL) has been applied extensively to differentiate the severity, classification and prognosis of periodontitis, to establish a periodontal treatment plan and to evaluate the results of dental treatment. Most periodontists measure 6 positions per tooth to determine the amount of CAL and elucidate 3-D alveolar bone structure by 2-D periapical X-ray findings of a tooth [15]. Complicated root anatomy, including an irregular cross-section shape, cervical enamel projection, enamel pearls, bifurcation ridges, root concavities, developmental grooves, hypercementosis, root proximity and furcation involvement, can affect the measurement of periodontal attachment [16]. However, detailed information on the association between the 2-D periodontal attachment loss (clinical attachment levels and height of radiographic bone loss vs. total root length) and the 3-D RSA detachment (the lost amount of connective tissues and bone attachment vs. total root surface area) is limited.

Maxillary premolars demonstrated a significantly shorter root length and a higher RSA than mandibular premolars, which produced results similar to those produced by the methods that involve applying a dental laser scanner [17]. Essentially, the first and second premolars that were collected in this study presented dental roots that were anatomically similar (Tomes’ root trait grade 0–2), which suggests that the related statistical discrepancy can be decreased [18]. Alternatively, the present study employed a micro-CT to analyze the samples according to the methods of an analogous micro-CT study to determine the smoothness of the models using software in order to avoid the roughness factor of dental root, which can greatly influence the amount of root surface area [18]. Furthermore, the dental laser study mainly examined the percentage of bone and periodontal RSA attachment loss according to McGuire’s classification [19–23]; this survey focused on determining the CAL to RSA loss in mm by following the APP guidelines for determining the severity of periodontitis [1]. Instead of correlating radiographic 2-D bone-supported root length with RSA, this study aimed to examine the lost amount of RSA and the percentages at the evaluated PALs in mm corono-apically from the CEJ to the 10^th^ mm. Theoretically, micro-CT possesses higher spatial resolution (15–18 μm) than a dental laser scanner (20 μm); however, compatible results have been obtained. Therefore, both dental laser scanners and micro-CT are equal in surveying the tooth’s surface. A different long axis of force distribution on maxillary or mandibular premolars, less maxillary bone density, and the anatomic position of sinus and nasal cavities may partially explain why maxillary premolars result in a significantly higher RSA and shorter root length.

Both maxillary and mandibular premolar roots are shielded with approximately 11.5% RSA at the 1^st^ mm PAL apical to the CEJ. The proximal root concavity and the CEJ position at diverse horizontal levels (corono-apically) partially explains why there is more RSA/mm at the 1^st^ mm PALs for both premolars. After that, close to 10% RSA/mm was retained coronally at the 2^nd^ to 4^th^ mm PAL for maxillary premolar roots vs. the 2^nd^ to 3^rd^ mm PAL for mandibular premolar roots; approximately 9% RSA/mm covered the 4^th^ to 6^th^ mm PAL for maxillary premolar roots vs. the 4^th^ to 5^th^ mm PAL for mandibular premolar roots; 8% RSA/mm was retained at the 7^th^ to 8^th^ mm PAL for maxillary premolar roots vs. the 6^th^ to 8^th^ mm PAL for mandibular premolar roots; 7% RSA/mm sheltered the 9^th^ mm PAL for maxillary and mandibular premolar roots; and 5.5% RSA/mm for maxillary premolar roots vs. 6.6% RSA/mm for mandibular premolar roots at the 10^th^ mm PAL. An insignificant difference in RSA per mm was noted at all corresponding PALs except the 2^nd^ to 3^rd^ mm PAL for maxillary and mandibular premolars (Table 2). A divergent tendency and a sandglass cross-sectional shape of maxillary premolar root anatomy somewhat explained the difference that was found at the 2^nd^ to 3^rd^ mm PAL. Otherwise, taper premolar roots supported the findings of decreasing RSA corono-apically. Generally, the taper effects of premolar roots according to RSA were insignificant by the 1-mm PAL measurement (Table 3); however, the taper effect of premolar roots became obvious when the RSA analysis was carried out every 2 mm (Table 4).

The findings of this RSA investigation may help associate the concept of biologic width that includes 1.07 mm connective tissues and 0.97 mm epithelium [24]. Whether it was epithelium, connective tissues or bone attachment, approximately 10% RSA/mm covered the coronal 4 mm of maxillary premolar root surfaces and 3 mm of mandibular premolar root surfaces. Once the alveolar bone level retrogresses more than 1mm apically, the premolars lost 10% RSA/mm bone support at coronal 3 to 4 mm levels in premolars, 9% RSA/mm at the 5^th^ to 6^th^ mm of maxillary premolar roots and at the 4^th^ to 6^th^ mm of mandibular premolar roots (Table 2). Under the premise that root length can be accurately measured radiographically, 9% to 10% RSA/mm discrepancies exist between the radiographic bone level and clinical attachment level from the 2^nd^ to 6^th^ mm corono-apically without taking epithelium attachment into account.

We refer to the AAP guidelines for determining the severity of periodontitis; the guidelines define mild periodontitis as up to 15% of root length or ≥2mm & ≤3mm of radiographic bone loss [1]; this 3-D observation presented some inconsistent findings, such as the RSA study demonstrating that ≤2 mm instead of ≤3 mm (in AAP) of radiographic bone loss was concurrent with the position at 15% of root length and that 21% RSA instead of 15% RSA corresponded to the level of 15% RBL. Additionally, when taking 1 mm of connective tissue attachment into account (approximately 10% RSA), approximately 1 mm of clinical attachment loss rather than 1 to 2 mm CAL was associated with mild periodontitis. Similarly, 4 mm instead of ≤5mm of radiographic bone loss corresponded to the crestal position at 30% RBL; and there was approximately 40% coronal bone supported RSA loss as opposed to 30% bone supported RSA loss corresponding to a crestal level of 30% RBL. Additionally, if 1 mm of connective tissue attachment was included (approximately 9–10% RSA), it converted into approximately 3 mm of clinical attachment loss rather than 4 mm of CAL characterizing moderate periodontitis (Table 3). Moderate periodontitis translated easily into severe periodontitis when the RSA condition was considered. Moreover, this RSA study displayed that there was approximately 50% periodontal detachment when PAL was at the 5^th^ mm, and more than 60% bone supported RSA was lost when the crestal bone level was at the 50% RBL. The anchored quality of epithelium attachment was incompatible with the connective tissue attachment. In addition, once the condition of epithelium attachment was taken into consideration, the RSA discrepancy between the examined PAL levels and the bone-supported RSAs was enhanced; therefore, the epithelium attachment discussion was not included in this study. Diverse root length, size, morphology and taper partially explain the inconsistency between this RSA study and the AAP guidelines. For instance, the average root length in this premolar study is 12.6–13.3 mm, and the root length acquired in the APP guidelines is 16.7–20 mm (these numbers were conjectured from 15% radiographic bone loss, indicating a 3 mm root length and 30% radiographic bone loss, which indicates a 5 mm root length in Table 1 of the APP guidelines) [1]. In this study, only single-rooted premolars were examined, which is a factor of concern that may have affected the results. One previous study demonstrated that various tooth types remain dissimilar in supporting tissues at the same clinical attachment level. For example, a previous study reported that when an 8 mm CAL was detected for maxillary canines, approximately 43% of the supporting tissues remained. Conversely, a secondary maxillary premolar might only have 27% of its supporting tissues present at the same attachment level [9]. Another dental laser scan study supported the concept of the amount of decrease of supporting alveolar bone, which indicated that periodontitis at single-rooted premolars is underestimated by 2-D examinations because of the tapering effects of root shape [17]. However, only the teeth with a cylinder root (tapering angle = 0) can theoretically meet the consistency of 2-D and 3-D measurements. A more tapered root presents more of an RSA distribution at the coronal levels and more discrepancy between the 2-D and 3-D measurements. Both the root taper and the complexity of the root shape can contribute to the inconsistency between the two methods.

## Conclusion

Even with the limitations of this study, the results showed that approximately 10% RSA/mm is associated with a coronal 3–4 mm root length of premolars. The 3-D RSA amount measured every 2 mm decreased significantly along with the tapering of the roots from the CEJ to the apex. Therefore, premolars could lose approximately 10% of their RSA attachment corresponding to 1 mm of clinical attachment loss from mild to severe periodontitis. Sampling the periodontal microbial pathogens using information from 2-D radiographic bone and clinical attachment losses without taking the 3-D RSA into account could potentially lead to misinterpretation of the association between bacteria and periodontitis.

## Supporting information

S1 Table The RSA amount and percentage from 1^st^ mm to 10^th^ mm PALs of the maxillary and mandibular premolars. (XLSX)
