# Correction: Incompatible amount of 3-D and 2-D periodontal attachments on micro-CT scanned premolars

**DOI:** 10.1371/journal.pone.0209206

**Published:** 2018-12-10

**Authors:** Hsiang-Hsi Hong, Adrienne Hong, Yi-Fang Huang, Heng-Liang Liu

The incorrect image appears as Fig 1. Please see the correct Fig 1 here.

**Fig 1:**
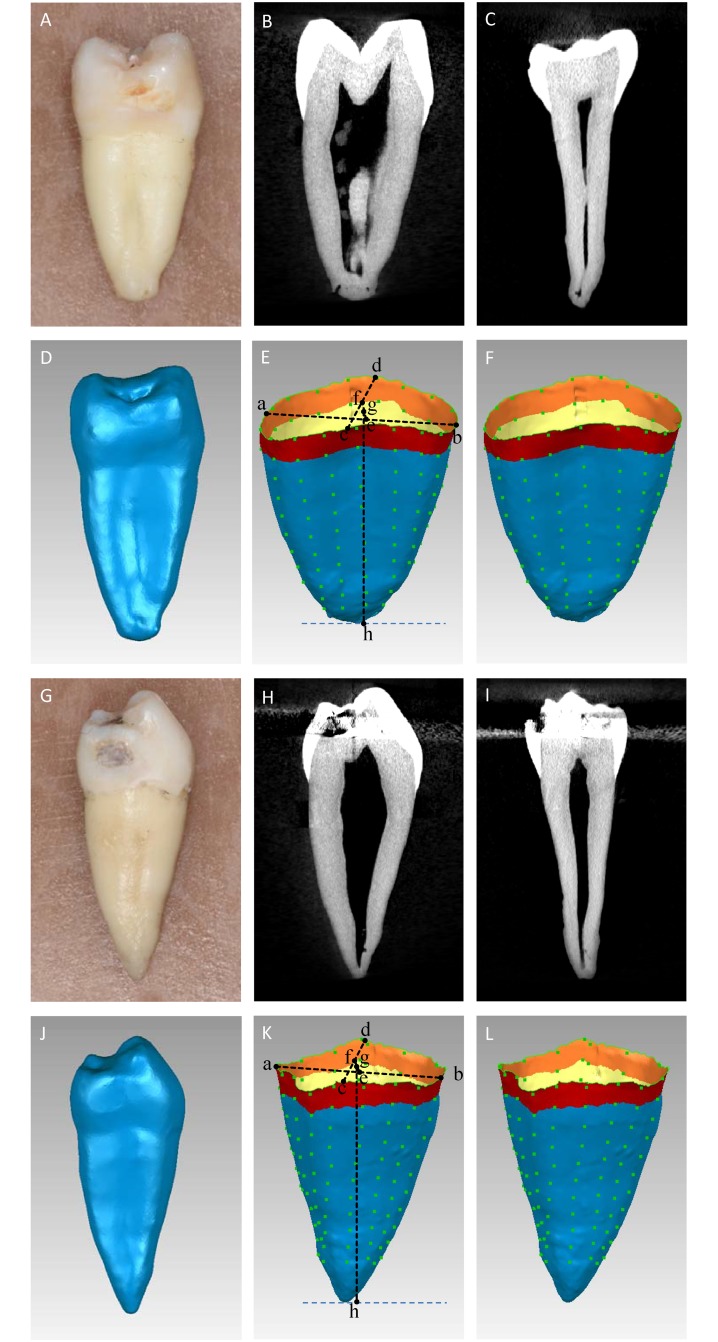
Photograph, micro-CT and STL images of scanned maxillary and mandibular premolars. (A–F): The views and estimated levels of a micro-CT scanned maxillary premolar. (A): maxillary premolar; (B): a sagittal view; (C): a frontal view; (D): a STL format model was developed.; (E): a–b: line connecting the buccal and lingual CEJ, c–d: line connecting the mesial and distal CEJ, e: midpoint of a–b, f: midpoint of c–d, g: midpoint of e–f and represented the CEJ from 2–D viewpoint, and g–h: represented the 2–D root length; (F): The root surface areas were calculated from 1^st^ mm to 10^th^ mm. (G–L): The views and evaluated levels of a micro-CT scanned mandibular premolar. (G): mandibular premolar; (H): a sagittal view; (I): a frontal view; (J): a STL format model was developed; (K): a–b: line connecting the buccal and lingual CEJ, c–d: line connecting the mesial and distal CEJ, e: midpoint of a–b, f: midpoint of c–d, g: midpoint of e–f and represented the CEJ from 2–D viewpoint, and g–h: represented the 2–D root length; (L): The root surface areas were calculated from 1^st^ mm to 10^th^ mm corono-apically.

## References

[1] Hong H-H, Hong A, Huang Y-F, Liu H-L (2018) Incompatible amount of 3-D and 2-D periodontal attachments on micro-CT scanned premolars. PLoS ONE 13(3): e0193894 10.1371/journal.pone.0193894 29518113 PMC5843242

